# Bayesian assignment of gene ontology terms to gene expression experiments

**DOI:** 10.1093/bioinformatics/bts405

**Published:** 2012-09-03

**Authors:** P. Sykacek

**Affiliations:** Department of Biotechnology, BOKU University, Muthgasse 18, 1190 Vienna

## Abstract

**Motivation:** Gene expression assays allow for genome scale analyses of molecular biological mechanisms. State-of-the-art data analysis provides lists of involved genes, either by calculating significance levels of mRNA abundance or by Bayesian assessments of gene activity. A common problem of such approaches is the difficulty of interpreting the biological implication of the resulting gene lists. This lead to an increased interest in methods for inferring high-level biological information. A common approach for representing high level information is by inferring gene ontology (GO) terms which may be attributed to the expression data experiment.

**Results:** This article proposes a probabilistic model for GO term inference. Modelling assumes that gene annotations to GO terms are available and gene involvement in an experiment is represented by a posterior probabilities over gene-specific indicator variables. Such probability measures result from many Bayesian approaches for expression data analysis. The proposed model combines these indicator probabilities in a probabilistic fashion and provides a probabilistic GO term assignment as a result. Experiments on synthetic and microarray data suggest that advantages of the proposed probabilistic GO term inference over statistical test-based approaches are in particular evident for sparsely annotated GO terms and in situations of large uncertainty about gene activity. Provided that appropriate annotations exist, the proposed approach is easily applied to inferring other high level assignments like pathways.

**Availability:** Source code under GPL license is available from the author.

**Contact:** peter.sykacek@boku.ac.at

## 1 INTRODUCTION

The well-known gene ontology (GO) (Ashburner *et al.*, 2000) is at the center of different research questions in Systems Biology and Bioinformatics. Even for well-studied model organisms such as *Saccharomyces cerevisiae*, annotations of genes to ontology terms is far from complete. To improve this situation, (de Queiroz *et al.*, 2006) proposed a BLAST (Altschul *et al.*, 1990, 1997)-based sequence similarity analysis and machine learning approaches for a *denovo* annotation of genes to GO terms. Novel-scientific discoveries require a constant updating of the standardized gene ontology (Ashburner *et al.*, 2000). Maintaining a well-curated specification is a tedious and time-consuming manual task. For improving the reaction time (McGarry *et al.*, 2007) propose inferring Bayesian networks by literature mining to generate domain-specific ontologies automatically. With the purpose of benchmarking gene associations inferred with Bayesian networks, (Troyanskaya *et al.*, 2003) propose using known GO term annotations for assessing the significance of inferred associations.

Research is also concerned with mapping biological assays like expression experiments to global biological function. Such approaches use known gene to GO annotations and represent biological function by assigning standardized GO terms to experimental data. Recent surveys (Dopazo, 2006; Huang *et al.*, 2009; Khatri & Drahici, 2005) show that a majority of these methods use statistical test-based inference. Strategies are often modular and will first rank genes using state-of-the-art expression data analysis which includes statistical test based (Pan, 2002; Reiner *et al.*, 2003; Tusher *et al.*, 2001; Wernisch *et al.*, 2003), and Bayesian methods (Bae & Mallick, 2004; Lee *et al.*, 2003; Li *et al.*, 2002; Posekany *et al.*, 2011; Sykacek *et al.*, 2007) More recently, the quantification of transcript abundance from next generation sequencing data (Guttman *et al.*, 2010; Trapnell *et al.*, 2010) attracted a lot of attention.

Approaches like FatiGO (Al-Shahrour *et al.*, 2004) and Onto-express (Draghici *et al.*, 2003*a*, *b*) rely on rank lists obtained from first level analysis, separate the genes which are annotated to GO terms into groups and use Fishers exact test, the hyper geometric distribution or similar approaches for calculating *P*-values of GO term enrichment. All GO terms with *P*-values below a suitably chosen threshold are then assigned to the experiment, with the inherent multiple testing problem being tackled with state-of-the-art approaches. Separation into lists of active and inactive genes depends greatly on the chosen threshold. As is illustrated in (Dopazo, 2006), in particular conservative thresholds are likely to underestimate over-representation of GO terms by functionally active genes. A solution to this problem was suggested as Fati-Scan (Al-Shahrour *et al.*, 2006), which repeatedly applies FatiGO with different thresholds, adjusts *P*-values correspondingly and thus reduces the effect of choosing a *particular* threshold. With BayGO, (Vencio *et al.*, 2006) proposed a Bayesian tool for inferring active GO terms. BayGO is based on gene counts allows, however, for unobserved genes which may result from missing probes or poor quality measurements. The approach regards the true number of active and inactive genes as random variables and infers active GO terms by calculating significance levels of (lack of) association via Monte Carlo simulation. If all annotated genes are observed, the method will provide the same result as statistical tests based on the hyper geometric distribution. More recently, (Zhang *et al.*, 2010) proposed another Bayesian approach for counts-based GO term enrichment which considers the GO DAG structure to ease identification of groups of closely related GO terms.

Arecent investigation in (Huang *et al.*, 2009) finds methodological biases in the assigned GO terms which depend on the statistical approach used for assessing significance. Their observation is likely caused by variations in the power of the tests which in general increases with sample size. Inferring active GO terms with a statistical test will thus inevitably favor GO terms which represent well-studied concepts with many annotated genes.

Counts-based GO term enrichment analysis has in addition the disadvantage of considering all genes on one side of the threshold equally, irrespective of the assigned significance level or posterior probability of functional importance. Essential information from first level data analysis about the degree of believe we should have about gene activity gets thus removed.

A thoroughly Bayesian GO term inference should, however, consider the uncertainties about gene importance which we get from first level data analysis. The Bayesian approach we propose in this article considers this uncertainty by representing every gene as a binary random variable. This requires modeling expression data such that gene wise posterior probabilities quantify the degree of believe we have about gene activity. The essential difference between our proposed analysis and the Bayesian methods in (Vencio *et al.*, 2006; Zhang *et al.*, 2010) is that we consider the uncertainties about gene activity during GO term inference. GO term inference is here implemented by a probabilistic model which has some similarity with clustering methods. Using the proposed approach, we may expect avoiding biases toward GO terms with many annotated genes: even sparsely annotated GO terms will be assigned to an experiment with high probability, if all assigned genes are with high probability found to be expressed.^1^ By combining the probabilities about expression of individual genes, probabilistic GO term inference considers thus additional information which is discarded by a threshold-based enrichment analysis.

After providing an overview of the proposed approach in the ‘Methods’ section, we discuss several applications of the proposed approach. Inferring hypothetical GO terms from synthetic data illustrates advantages we may expect from probabilistic GO term inference over counts-based GO term enrichment methods. GO term inference for microarray data is used for further investigations. A previously published (Sykacek *et al.*, 2007) probability measures of shared gene function in (i) a cycle of mouse mammary gland development and (ii) the process of *in vitro* endothelial cell apoptosis obtains with the proposed approach a stronger enrichment of cell death-related GO terms at the top of the rank list, as was found in Ref. (Sykacek *et al.*, 2007) with inference based on Fisher's exact test. Finding cell death-related GO terms is in line with previous reports that apoptosis of endothelial cells is known to occur during the mammary gland cycle and may play an important role in this process (Djonov *et al.*, 2001; Matsumoto *et al.*, 1992). In a second experiment on the mRNA expression dataset from (Castells-Roca *et al.*, 2011), a heat shock experiment in *S. Cerevisiae*, we obtain very little uncertainty about gene activity. Even in this situation where we do not expect advantages when considering these uncertainties, a counts-based enrichment analysis is at best equally well suited than the proposed method. We may, therefore, conclude that the moderate increase in computation of the probabilistic GO term analysis over a counts-based approach is time well spent. Bayesian GO term inference is a viable alternative to existing GO term enrichment methods, in particular, if the expression data was already analyzed with a compatible Bayesian method.

## 2 METHODS

### 2.1 Probabilistic model for inferring active GO terms

**Fig. 1.**
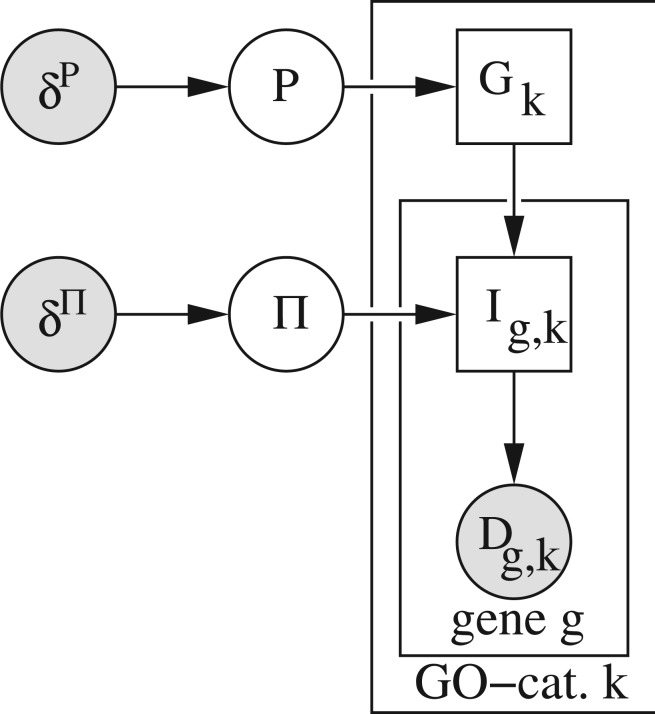
A directed acyclic graph (DAG) representing GO term inference. The graph represents discrete variables by rectangles and continuous quantities as circles. Plates (or sheets) denote repeated conditional independence relations. Shaded nodes are observed variables or specified quantities. The clear nodes represent random variables which are subject to inference. GO terms are represented as binary indicator variables, *G*_k_, with *G*_k_ = 1 indicating active and *G*_k_ = 0 dormant terms. The binary indicators *I*_g,k_ represent genes which are assigned to the *k*-th GO term. Genes which we find active in the corresponding microarray data *D*_g,k_ are represented by *I*_g,k_ = 1, whereas *I*_g,k_ = 0 indicates inactive genes. The prior probability of GO terms being active is modeled hierarchically as Beta-Bernoulli model, using *δ^P^* = {*δ*_*G*_*k*__^*P*^; ∀G_*k*_} as prior counts and *P* for denoting the prior probability. The conditional probabilities of observing active genes are modeled by Beta-Bernoulli models as well. We use Π = {Π_0_ = *P*(*I*_g,k_|*G*_k_ = 0), Π_1_ = *P*(*I*_*g, k*_ |*G*_*k*_ = 1)} as conditional probabilities and *δ*^Π^ = {*δ*_*G*_*k*_, *I*_*g,k*__^Π^;∀**G**_*k*_, *I*_*g, k*_} as corresponding prior counts

We propose inferring the activity of GO terms by the probabilistic model illustrated in Figure 1. GO terms are represented as binary indicator variables, *G*_k_, where *G*_k_ = 1 implies activity and *G*_k_ = 0 a dormant term. Activity is inferred by relating the indicator variable of the *k*-th GO term, *G*_*k*_, to all genes with known annotation to that GO term. Variable *I*_g,k_ is again a binary indicator variable, with *I*_g,k_ = 1 indicating activity of the corresponding gene and *I*_g,k_ = 0 a lack thereof. The probability whether a gene is active or not depends on the corresponding expression data *D*_g,k_. The graphical model in Figure 1 denotes the prior probabilities of GO term activity by variable *P*, with *P*(*G*_k_ = 1|*P*) denoting the prior of GO term activity and *P*(*G*_k_ = 0|*P*) the prior of being dormant. Variable Π represents the conditional probabilities *P*(*I_g,k_* |*G_k_*) which determine the probabilities of observing active and inactive genes for active and dormant GO terms.

In principle, we could fix both probabilities. Ignorance about the activity of GO terms were then coded by *P*(*G_k_* = 1|*P*)= 0.5. Our intention that an active GO term should with high-probability correspond to observing active genes were coded by *P*(*I*_g,k_ = 1|*G*_k_ = 1)= 1. We could as well assume that a dormant GO term implies with high-probability inactive genes and set *P*(*I*_g,k_ = 0|*G*_k_ = 0)= 1. Inferring active GO terms is then a message passing problem and requires applying Bayes theorem and normalization. A disadvantage of this approach is its inadequacy in situations where not all genes annotated to an active GO term are active and in the converse situation, where not all genes annotated to an inactive GO term are necessarily inactive.

To cope with more realistic impure situations, we suggest adding an additional hierarchy to the model and inferring the variables *P* and Π as well. This is achieved by including a beta prior over *P* and Π_*G*_*k*__ with prior counts *δ^P^* = {*δ*_G_k__^*P*^; ∀G_*k*_} and *δ*^Π^ = {*δ*_G_k_, *I*_g,k__^Π^;∀*G*_*k*_, *I*_g,k_}. This results in a fully probabilistic approach, with *I*_g,k_ being modeled as a two-component mixture of Bernoulli distributions. In addition, *P* and Π are random variables and thus part of the inference problem. Without taking further steps, this model does not allow the indicator *G*_k_ to be fully identified as inference does not determine whether *G*_k_ = 1 or *G*_k_ = 0 codes for active GO terms. Using the preconception formulated above, that active GO terms are those having a larger number of active genes annotated to, requires thus after inference to identify the solution once.

### 2.2 Parameterizing the joint density

Parameterizing the joint density represented by the DAG in Figure 1 requires specifying all conditional densities, where we use *l* = [1, 2] in all equations below for indexing the two states of the binary indicators (*G*_k_ and *I*_g,k_) and the corresponding count values in the Beta densities. The prior over *P* is modeled as a Beta density
(1)
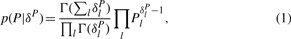

with Γ(*δ_l_^P^*) denoting a gamma function. Similarly, we get as prior over Π a product of Beta density functions
(2)
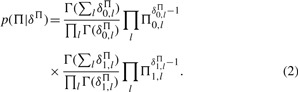

The conditional densities of *G*_k_ and *I*_g,k_ are Bernoulli densities
(3)
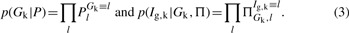

The specification of the model is completed by *p*(*D*_g,k_|*I*_g,k_) which denotes the marginal likelihood of the data given the gene indicator. Using *γ*_k_ for denoting the number of genes annotated to GO term *k*, we finally obtain the joint density implied by the graphical model in Figure 1 as
(4)
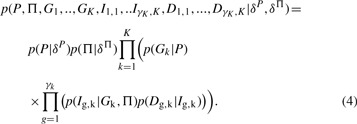


### 2.3 Marginal likelihoods from probabilistic microarray analysis

The marginal likelihoods *P*(*D_g,k_* |*I_g,k_*) in the model specification can be obtained from all probabilistic approaches to microarray data analysis, which provide posterior probabilities of gene function as a result. We can, for example, use the marginal probabilities over gene indicators as they arise from Bayesian variable selection in (Bae & Mallick, 2004; Lee *et al.*2003) or the posterior probabilities of shared gene function proposed in (Sykacek *et al.*, 2007). Using the above notation, the information obtained by Bayesian expression data analysis about genes is typically summarized by a probability measure *P*(*I*_g,k_|*D*_g,k_), with the probability that the corresponding gene is involved in the biological assay being given as *P*(*I*_g,k_ = 1|*D*_g,k_).

By applying Bayes theorem, these probabilities can be converted to quantities which are proportional to marginal likelihoods
(5)
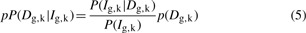

with the marginal likelihood *P*(*D*_g,k_|*I*_g,k_ = 1) arising from a model having the corresponding gene active and *p*(*D*_g,k_|*I*_g,k_ = 0) being the marginal likelihood if the gene is dormant. The multiplicative constant *p*(*D_g,k_*) in Equation (5) is independent of *I*_g,k_ and cancels out during inference of the graphical model in Figure 1. This suggests that we can replace the marginal likelihoods *P*(*D*_g,k_|*I*_g,k_) with posterior probabilities of gene activity divided by the corresponding prior probabilities. We can thus use the proposed method as post-processing step to all Bayesian methods which calculate measures of gene activity, *P*(*I*_g,k_|*D*_g,k_), for gene expression assays.

### 2.4 Variational inference

Inferring active GO terms require calculating the marginal posterior distributions over all *G*_k_ indicator variables in the DAG in Figure 1. For reasons of tractability, we follow previous examples in the bioinformatics community (Beal *et al.*, 2005; Sykacek *et al.*, 2007) and resort to variational methods for inference. Variational approximations (Attias, 1999; Frey, 1998; Jordan *et al.*, 1999) are computationally efficient. This comes, however, at the price of introducing systematic approximations to the posterior distribution of GO term activity, *P*(*G*_k_|*D*_1,k_, .., *D*_*γ*_*k*_, k_, *δ^P^, δ*^Π^).

We will first define some abbreviations which ease mathematical notations below and use ***θ*** = {*P,* Π,* G*_1_,* .., G_K_*, *I*_1,1_, ..*I*_*γ_K_*_ *K* } for denoting all random variables in the DAG in Figure 1 and 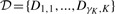 for abbreviating all data. Variational learning requires approximating the joint distribution of the model in Equation (4) by a factorising Ansatz
(6)
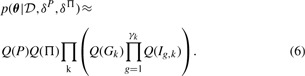

Jensen's inequality allows obtaining a lower bound on the log marginal likelihood of the DAG.
(7)
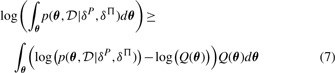

By integrating the second line in Equation (7) w.r.t. ***θ***, i.e. w.r.t. all random variables of the model, we obtain a quantity which is called the *negative free energy* of the model. Its main use in the proposed implementation is for diagnosis purposes and for assessing convergence of the approximation. Variational learning requires maximizing the lower bound in Equation (7) w.r.t to all *Q*-distributions. By integrating this bound with respect to all but one *Q*-distributions from Equation (6) and maximizing the resulting functional with respect to the remaining *Q*-function, we obtain for every *Q*-function in Equation (6) a separate update rule. These updates are done iteratively, until the negative-free energy converges. The most important result of the algorithm are the *P*(*G_k_* |*D*)≈*Q*(*G_k_*);∀; *k*, i.e. all approximate marginal distributions representing the posterior probabilities of GO term activity. What remains is deciding about which GO terms we should report as active. This depends on the relative cost we assign to false-positive and false-negative GO terms. If both errors are equally expensive, the Bayes optimal decision is assessing GO term *k* as active, if *P*(*G_k_* = 1|*D*)*>* 0.5 and otherwise declare it as inactive.

### 2.5 Algorithms for probabilistic GO analysis

To infer GO term probabilities from expression experiments, we perform the following steps.

#### 2.5.1 Expression data analysis

To be able to use the proposed method, we have to infer posterior probabilities of gene function from the expression data under consideration. For microarrays, such analysis can, for example, be done with the approaches proposed in Ref. (Bae & Mallick, 2004; Lee *et al.*, 2003; Posekany *et al.*, 2011; Sykacek *et al.*, 2007). Inference requires a careful sensitivity analysis with multiple runs started with different initial conditions. When relying on variational approximations for obtaining posteriors, the overall analysis can be done within about half an hour of runtime.

#### 2.5.2 Preparing GO annotations

As a second prerequisite to the proposed approach, we have to convert gene-to-GO-term annotations for the chosen organism to a format which is suitable for inference. For maintaining consistency, we annotate every gene not only to the original GO term, but also to all ancestral terms until the highest ancestral term with an existing manual gene annotation was reached. We get, therefore, annotations which reflect the term relationships in the GO DAG. Because we do only traverse upwards to levels where human annotations have previously been made, this approach generates a custom level of abstraction (Khatri & Drahici, 2005) which warrants that inference is of biological interest. The annotated GO DAG is then written into a tabulator delimited file which contains the GO terms in the first column and all annotated genes in the corresponding rows.

#### 2.5.3 GO term inference

After these preparatory steps, we calculate for all annotated GO terms the posterior probability of relevance to the underlying experiment. These probabilities are obtained by inferring the approximate marginal distributions over all random variables in the DAG in Figure 1. The free parameters in our GO term inference are the prior counts, *δ^P^* and *δ*^Π^. For specifying uninformative prior counts^2^, we set *δ^P^* and *δ*^Π^ to 1. Note that this prior setting does not guarantee that *G*_k_ = 1 implies GO term activity. We have thus got to identify the inference result once after inference is completed and possibly exchange the parameters of the posterior such that GO term assignment is coded by *G_k_* = 1. The algorithm shares some similarity with clustering, which implies that it finds a locally optimal mode. We should thus repeat inference several times from randomly chosen starting points for making sure that a suitable solution was found. Different solutions can be judged by their negative-free energy, with the optimal solution having attained the largest value. One inference run will typically take between one and 3 min. Within at most half an hour, we can thus iterate 10 inference runs, which we found so far sufficient for obtaining meaningful GO term assignments. As a result from inference, we store GO terms and the approximate posterior *P*(*G_k_* = 1|*D*), that is the probability that the term is active, into a tab delimited file. For typical datasets, the overall time required for first level data analysis and the proposed Bayesian GO term inference will be around 1 h run time on a conventional personal computer, which clearly demonstrates the feasibility of the approach.

### 2.6 Data

**Fig. 2.**
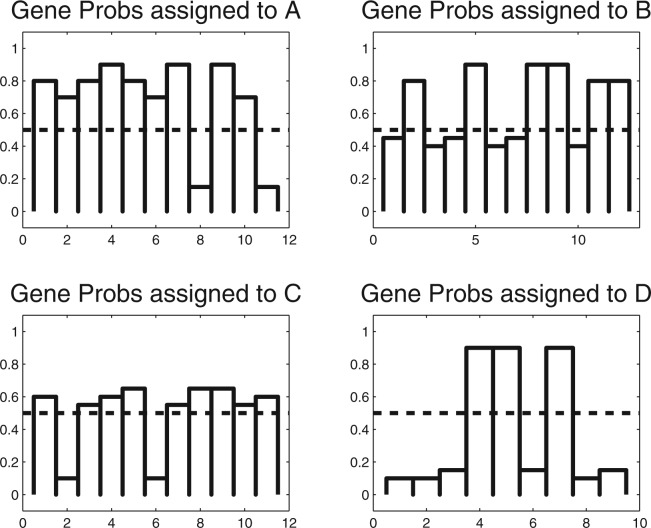
Figure showing four sub-plots illustrating hypothetical probabilities of gene activity. The dashed line indicates the threshold probability of 0.5 used in determining the counts of active versus inactive genes in Fisher's exact test. The genes assigned to the four hypothetical GO terms have different probabilities of being active. Term ‘A’has a large majority of highly active genes assigned to. Half of the genes assigned to term ‘B’ are highly active whereas the rest is ‘just not active’. Term ‘C’ has, like term ‘A’ a majority of active genes assigned to. The probabilities are, however, just above 0.5 and two genes are with high probability assessed as inactive (low probability for being active implies high probability for being inactive). The majority of genes assigned to term ‘D’ are with high probability inactive. Inference results are found in Table 1. A detailed discussion of the purpose of this experiment is provided in the main text

We analyze the proposed approach for GO term inference with synthetic data and two microarray datasets. Synthetically generated data are first used for contrasting the behavior of the proposed Bayesian GO term inference with classical enrichment analyses. Figure 2 illustrates for that purpose four hypothetical GO terms, each of which is annotated with genes we find expressed with different probabilities. A second synthetic test case was generated to assess the entire analysis pipeline from log expression measurements to assigned GO terms. We generate for that purpose 2000 hypothetical GOterms, each of which is annotated by between 1 and 100 genes. With 20% chance a GO term is declared ‘active’. For active GO terms, we assume observing differentially expressed genes with a chance of 80%. For inactive GO terms, we assume a 20% chance of observing differentially expressed genes. Data are generated to mimic a two-level experiment with log expressions of differentially expressed genes drawn from two Gaussians with mean +1 and –1. Log expressions of non-differentially expressed genes are drawn from zero mean Gaussians. For investigating the influence of differential expression uncertainty, we generated two datasets: one experiment generated the log expression data using a Gaussian with a standard deviation of 0.6; the other experiment used a Gaussian with a standard deviation of 1.2. Data were generated such that we obtain 20*10^3^ hypothetical genes and eight samples per group. Because we know which GO terms we should assign to each experiment, we can compare the accuracy of the proposed method with the accuracy of a classical counts-based enrichment analysis.

The synthetic investigations are complemented with analyses on microarray data. One analysis is based on indicator probabilities of shared gene function that were previously reported in Ref. (Sykacek *et al.*, 2007). These probabilities are obtained from an analysis of (1) a cycle of growth and regression in mammary glands *in vivo* (Clarkson *et al.*, 2004) and (2) an assay of programmed endothelial cell death investigated *in vitro* (Johnson *et al.*, 2004) for shared gene function. As a second biological experiment, we chose the mRNA expression dataset from (Castells-Roca *et al.*, 2011), a heat-shock experiment in *S. Cerevisiae*. The genes in both datasets were mapped to a recent version of the GO DAG, which is available from http://www.geneontology.org.

## 3 RESULTS

We obtained all results which we report here with the algorithmic settings that were proposed in the ‘Methods’ section. This section compares the proposed probabilistic analysis for inferring active GO terms with a classical counts-based approach. Classical inference is based on Fisher's exact test which is mainly motivated by its popularity and availability in many analysis packages (Dopazo, 2006; Khatri & Drahici, 2005).

### 3.1 Hypothetical GO term inference

The purpose of a hypothetical GO term inference is illustrating the properties of the proposed analysis. We will in particular discuss two situations where the proposed probabilistic approach provides identical conclusions as inference with Fisher's exact test. For two other examples, the results about active GO terms differ. The synthetic experiment uses four hypothetical GO terms ‘A’to ‘D’. The posterior probabilities of hypothetical gene activity, which represent the *P*(*I*_g,k_|*D*_g,k_) in the derivation of GO term inference, are shown in Figure 2. Term ‘A’ has a large majority of highly active genes assigned to. Half of the genes assigned to term ‘B’ are highly active, whereas the rest is ‘just not active’. Term ‘C’ has, like term ‘A’ a majority of active genes assigned to. The probabilities are, however, just above 0.5 and two genes are with high probability assessed as inactive (low probability for being active implies high probability for being inactive). A majority of genes assigned to term ‘D’ are with high probability inactive.

The result in Table 1 shows that the probabilistic assessment and Fisher's exact test assess term ‘A’ as active. They also agree about term ‘D’ which is found being inactive. The approaches do, however, differ about terms ‘B’ and ‘C’. Term ‘B’ has six genes assigned to which show a large probability of being active. The other six genes are found inactive with probabilities close to 0.5 indicating large uncertainty. Probabilistic inference combines these probabilities and concludes that this situation points with high probability to an active GO term. Statistical tests based on counts ignore these probabilities and will thus generate very large *P*-values for the null hypothesis. The different result for term ‘C’ is also caused from test-based approaches ignoring certainty levels. Although term ‘C’ has 10 genes assigned to, which are more likely active than inactive, the probabilities are just above 0.5, indicating large uncertainties. A counts-based test ignores these uncertainty levels and regards a gene as active and consequently assigns a significant enrichment with active genes. The probabilistic approach considers the uncertainty implied by small probabilities and combines these small probabilities, in favor of gene activity with two probabilities, which state the opposite with much more certainty. The result is that a situation as shown here for term ‘C’ leads to a large probability assessing inactivity of that GO term.

**Table 1.** Probabilistic versus. classical inference of hypothetical GO term activity

GO term	*P*(*G_k_* |*D*), Bayes	*P*-value, Fisher
Hypoth. A	1 (active)	0.033 (active)
Hypoth. B	1 (active)	0.61 (inactive)
Hypoth. C	0 (inactive)	0.033 (active)
Hypoth. D	0 (inactive)	0.91 (inactive)

The table displays for the Bayesian approach the probabilities that GO terms are active and for Fisher's exact test the *P*-value of the null hypothesis. In brackets, we indicate whether the GO term is assessed as active or inactive.

**Fig. 3.**
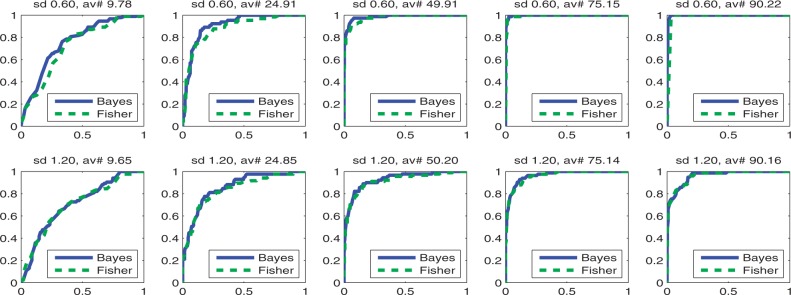
Graphs that allow comparing the performance of predicting known GO terms with the proposed Bayesian analysis to a classical count statistics-based enrichment analysis. We observe that larger noise levels (sd denoting the standard deviation) render inference of differential expression more difficult and consequently increase the difficulty of predicting GO term activity. This manifests itself in smaller areas under the ROC curves for all inferences which were obtained at the larger noise level. We can also deduce that inference gets easier for GO terms which are annotated with larger numbers of genes (av# denoting the average number of genes annotated to the GO terms) which again manifests in larger areas under the ROC curves we get for such GO terms. At the same time, the advantage for the proposed Bayesian GO term inference disappears for GO terms which have more genes annotated to, however, without leading to a situation that would disfavor the Bayesian approach for accuracy reasons

The second synthetic dataset is more realistic because we generate log gene expressions from known GO term activity states. The corresponding analysis comprises inferring differentially expressed genes and, based on these gene wise probabilities, inferring GO term activity. Probabilities of differential expression were obtained from a variational Bayesian analysis of variance (ANOVA) model which was implemented along the lines of (Posekany *et al.*, 2011). Because we know the state of every GO term, we can compare the results of both methods independent of thresholds with receiver operating characteristics (ROC curves). To simulate different branches in the GO DAG which are studied with different degrees of detail and thus annotated with different number of genes, we group the GO terms by the number of assigned genes. As thresholds we chose 16, 37, 63 and 84 genes. This results for both noise levels (std dev. of 0.6 and std dev. of 1.2) in five different ROC curves which we illustrate in Figure 3. As we can see, the Bayesian approach leads to larger areas under the ROC curve for GO terms which have fewer than about 40 genes annotated to. For larger numbers of genes annotated to GO terms, these differences disappear, without however leading to a situation where the Bayesian approach would be outperformed by the counts-based approach.

### 3.2 Inferring active biological processes from probabilities of gene function

#### 3.2.1 Apoptosis and differentiation in endothelial cells and mammary gland development

We will now turn to inferring active GO terms from the biological process subgraph of the gene ontology for two biological experiments. The first example uses the Bayesian probabilities that were calculated previously analysing two microarray time-course experiments: (1) a cycle of growth and regression in mammary glands *in vivo* (Clarkson *et al.*, 2004) and (2) an assay of programmed endothelial cell death investigated *in vitro* (Johnson *et al.*, 2004) for shared gene function. The results of this analysis were reported in Ref. (Sykacek *et al.*, 2007). Measurements were taken with Affymetrix arrays and genes cross annotated to human. The Affymetrix GO annotations were mapped to a recent version of the GO DAG. These initial steps provide indicator probabilities of shared gene activity and 2245 GO terms which we use as inputs for inferring GO term activity.

**Fig. 4.**
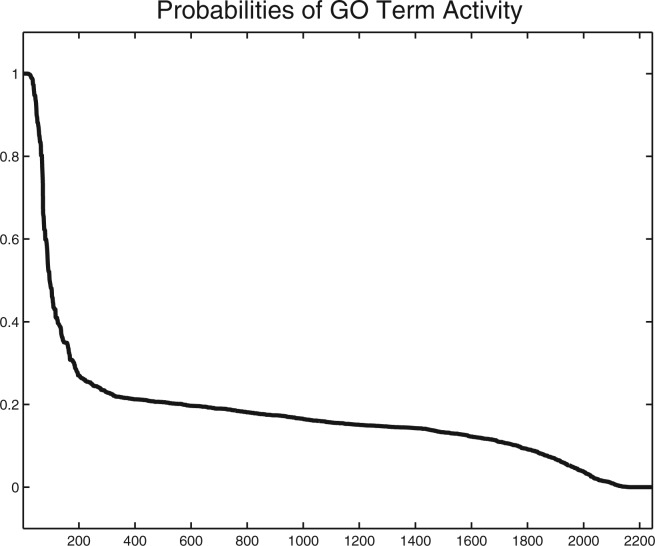
This figure shows probabilities of GO term activity, *P*(*G_k_* |*D*), ranked in decreasing order. GO term inference is based on indicator probabilities which assess *shared* gene activity. As a result, we find 95 active GO terms that have activation probabilities larger than 0.5. Assuming independence of GO term activity, the false discovery rate among these 95 genes is about 15%

The ordered posterior probabilities of GO term activity obtained from such inference are displayed in Figure 4. Assuming equal cost for false positives and false negatives, the Bayes optimal decision is to use probability 0.5 as lower threshold for assigning GO terms. This threshold provides 95 GO terms and, assuming independence of GO term activity, corresponds roughly to a false discovery rate (FDR) of 15%. Table 2 focuses attention to a smaller selection of GO terms that are most probably active. Using a conservative threshold of 0.995 (FDR *<* 1%) selects 27 GO terms. The GO terms in Table 2 are in decreasing order of probability of being active. GO term probabilities have been rounded for two digits after the decimal point and are consequently all 1. The list contains most notably five GO terms from the cell death subgraph. This is in line with previous findings that endothelial cell apoptosis may play an important role in mammary gland development (Djonov *et al.*, 2001; Matsumoto *et al.*, 1992). The observation that active GO terms from these indicator probabilities of shared gene function point to several cell death-related terms is in line with the Fishers exact test-based inference in (Sykacek *et al.*, 2007). The main difference is, however, that probabilistic GO term inference finds five such GO terms among the 27 highest probable GO terms and the test-based inference in (Sykacek *et al.*, 2007) finds as the highest ranked cell death related terms ‘induction of programmed cell death’ and ‘induction of apoptosis’ on positions 37 and 38. The proposed probabilistic approach shows thus in this example a stronger enrichment of expected GO terms at the top of the rank list.

**Table 2.** Table displaying active GO terms, we find from analyzing (1) a time course of mammary gland development and (2) a time course of endothelial cell apoptosis for shared gene activity

GO Id	Term name	*P*(*G*_k_|*D*)
0016043	Cell organization and biogenesis	1.0
0051649	Establishment of cellular localization	1.0
0051641	Cellular localization	1.0
0046907	Intracellular transport	1.0
0009058	Biosynthesis	1.0
0007242	Intracellular signaling cascade	1.0
0048523	Negative regulation of cellular process	1.0
0051243	Negative regulation of cellular physiological process	1.0
0015031	Protein transport	1.0
0043118	Negative regulation of physiological process	1.0
0008104	Protein localization	1.0
0045184	Establishment of protein localization	1.0
0044249	Cellular biosynthesis	1.0
0006886	Intracellular protein transport	1.0
0016070	RNA metabolism	1.0
0016192	Vesicle-mediated transport	1.0
0012501	Programmed cell death	1.0
0006915	Apoptosis	1.0
0008219	Cell death	1.0
0016265	Death	1.0
0044255	Cellular lipid metabolism	1.0
0006259	DNA metabolism	1.0
0006396	RNA processing	1.0
0006629	Lipid metabolism	1.0
0006412	Protein biosynthesis	1.0
0043067	Regulation of programmed cell death	1.0
0042981	Regulation of apoptosis	1.0

GO terms are in decreasing order of the assigned probability and included in the list, if the respective probability is >0.995. GO term probabilities have been rounded for two digits after the decimal point and are consequently all 1. The list contains most notably five GO terms from the cell death sub graph. This is in line with previous findings that endothelial cell apoptosis may play an important role in mammary gland development.

#### 3.2.2 Heat shock in S. cerevisiae

We will now briefly touch on a second biological experiment which was analysed for active biological processes. The study was recently published in Ref. (Castells-Roca *et al.*, 2011) and deposited at the gene expression omnibus under accession number GSE24484. The data are a time course with three control samples and five time points under heat-shock stress. The mRNA data comprises 18 expression arrays which were assessed using the GEO platform GPL4566. We used the first two time points (control before treatment and the first time point sampled 4 min after induction of heat stress) and selected those genes which allowed for an annotation to GO terms using the resources provided at http://www.geneontology.org. Data were vsn normalized (*cf.* (Huber *et al.*, 2002)) and probabilities of differential expression inferred using a variational implementation of the ANOVA model that was presented in Ref. (Posekany *et al.*, 2011). This lead to a small number of genes which were with large probability assessed as differentially expressed and a sharp transition to many genes which got very small probabilities, with uncertainty about involvement limited to very few genes. Very little uncertainty at the gene level suggests that the differences between counts based approaches and the proposed Bayesian method should for this data set be small. The proposed Bayesian approach for GO term inference was run for 10 times from random starting points. Predictions which were based on the solution which had the smallest free energy ranked the GO term ‘response to heat’ with probability 0.77 to position 81. To challenge the Bayesian approach, the counts-based approach was run several times with different thresholds leading to different sets of active and inactive genes. The different runs of counts-based GO term enrichment found ‘response to heat’ between positions 91 and up to position 80, that is, in the best case one position higher than the Bayesian solution.

The results suggest that the proposed Bayesian GO term inference has the potential to provide more accurate insights than a counts-based alternative. The Bayesian approach should in particular lead to favorable results for sparsely annotated GO terms and in situations where expression data analysis remains uncertain about gene activity for a large number of genes.

## 4 DISCUSSION

This article proposes a Bayesian approach for assigning GO terms to expression experiments which can be used as a post-processing step to Bayesian expression data analysis. Probabilities of GO term activity are obtained by combining results from probabilistic expression analysis in a Bayes' consistent manner. Calculations are isolated from pre- and post-processing and based on a tab delimited representation of GO term annotations and indicator probabilities which assess gene activity. Inference uses the variational Bayesian framework, which warrants that computations can feasibly be carried out on personal computers.

The results of the proposed approach are compared against a counts-based enrichment analysis which uses Fisher's exact test. Synthetically generated data reveals that the proposed Bayesian GO term assignment provides more accurate results for sparsely populated GO terms and in situations where expression data analysis has large uncertainty about gene activity. In scenarios, where a sufficiently large number of genes is annotated to GO terms and where expression data allow assessing genes activity with high probability, the Bayesian approach and counts-based enrichment analysis provide similar results, without, however, leading to situations which would disfavor the proposed Bayesian approach. Our assessment that GO term activity can be more reliably inferred by quantitatively combining probabilities of gene activity is also supported by analysing probabilities of shared gene function (Sykacek *et al.*, 2007) in mammary gland development (Clarkson *et al.*, 2004) and endothelial cell apoptosis (Johnson *et al.*, 2004). Compared with the GO term ranking obtained in Ref. (Sykacek *et al.*, 2007) with Fishers exact test, we observe with the proposed approach a stronger enrichment of cell death-related GO terms at the top of the GO term rank list. Shared apoptosis events in these assays are expected from reports that endothelial cell apoptosis may play an important role in mammary gland development (Djonov *et al.*, 2001; Matsumoto *et al.*, 1992). A second expression data set by (Castells-Roca *et al.*, 2011) which investigates heat shock stress in *S. cerevisiae* lead to a much more clear cut distinction between active and inactive genes. In this situation, we find a strong agreement between the proposed Bayesian GO term assignment and the counts-based enrichment analysis and thus equal performance.

The experiments allow hence the conclusion that a Bayesian GO term assignment has the potential of outperforming counts-based enrichment analysis in situations where GO terms are sparsely annotated and gene activity is difficult to assess. In situations with sufficiently many genes annotated to GO terms or with low uncertainty about gene activity, both counts-based enrichment and the proposed Bayesian assignment will provide similar accuracies. Although the application of the proposed Bayesian ontology assignment used gene ontology and microarray experiments as examples, the method is easily generalized to other ontology annotations and expression experiments by adapting the preprocessing filters and exchanging expression data analysis. The main limiting factor of Bayesian GO term assignment is the requirement of Bayesian indicator probabilities assessing gene activity. For statistical test-based array analysis, a comparable quantitative combination of *P*-values using statistical meta analysis can be obtained by applying the approach in Ref. (Gupta *et al.*, 2007).

## Footnotes

^1^‘Expression’ is used as synonym for a genes involvement in the studied biological experiment

^2^Specifying prior counts formalizes our prior belief of having observed a certain number of outcomes (here, for example, a certain number of active and inactive GO terms) *a priori*. In the light of the number of GO terms analyzed and the number of genes annotated to GO terms, prior counts below or equal to 1 are uninformative.
